# Effects of mindfulness-based interventions on perceived stress among non-clinical adults: a systematic review and meta-analysis

**DOI:** 10.1038/s44184-026-00188-4

**Published:** 2026-02-03

**Authors:** Anisha Rajan, Mahendra Kumar, Pranav Raj P

**Affiliations:** 1 Amity Institute of Behavioral and Allied Sciences, Amity University Chhattisgarh, Raipur, India; 2 Indian Institute of Technology Tirupati, Andhra Pradesh, India

**Keywords:** Diseases, Health care, Psychology, Psychology

## Abstract

Mindfulness-based interventions (MBIs) are recognised as effective psychosocial strategies for managing stress. We conducted a systematic review and meta-analysis of studies published up to August 2025 across the Web of Science, PubMed, Scopus, PsycINFO, EBSCO and Google Scholar following the PRISMA guidelines. Seventeen randomised controlled trials (*n* = 1,641) met the inclusion criteria. Baseline stress did not differ between MBI and control groups (SMD = 0.15, 95% CI = −0.07 to 0.36, *p* = 0.18). Post-intervention, MBIs were associated with significantly lower perceived stress (SMD = −0.53, 95% CI = −0.72 to −0.33, *p* < 0.00001). Within-group analyses indicated substantial reductions among MBI participants (SMD = 0.93, 95% CI = 0.62 to 1.23, *p* < 0.00001), whereas controls showed only marginal changes (SMD = 0.23, 95% CI = 0.00 to 0.46, *p* = 0.05). Subgroup analyses confirmed effectiveness across regions and delivery modalities, with larger effects observed in indirect interventions (SMD = −0.676, *p* < 0.001). Meta-regression found no significant moderators, and sensitivity analyses demonstrated robustness with minimal publication bias. Collectively, these results support MBIs as effective and scalable strategies for reducing perceived stress in non-clinical adults, highlighting the need for further evaluation of their effects on physiological stress markers.

## Introduction

Perceived stress is a key psychological concept that illustrates how individuals perceive and manage life’s challenges, independent of external stressors. It reflects subjective assessments of demands relative to coping resources and affects both mental and physical health outcomes^1,2^. High perceived stress has been associated with adverse effects, including anxiety, depression, cardiovascular problems, and changes in biological processes such as cortisol production and heart rate variability^3–5^. Due to these wide-ranging impacts, perceived stress has become a significant focus in both mental and public health research.

The Perceived Stress Scale (PSS), developed by Cohen, Kamarck, and Mermelstein in 1983, remains the most widely used tool for assessing subjective stress^6^. Based on the transactional model of stress proposed by Lazarus and Folkman. The PSS has demonstrated strong psychometric properties and is valued for its conciseness, reliability, and sensitivity to change^7,8^. Its repeated use across studies enhances comparability and makes it especially suitable for synthesising findings on stress interventions.

Mindfulness-based interventions (MBIs) have become prominent non-pharmacological approaches for stress reduction. Mindfulness is described as intentional, non-judgmental awareness of the present moment, encompassing thoughts, emotions, and bodily sensations^9^. MBIs are believed to reduce stress through mechanisms such as activating the parasympathetic nervous system, improving emotional regulation, and enhancing psychological flexibility^10^. Neurobiological evidence further suggests that mindfulness practice is associated with structural and functional changes in brain regions involved in emotional regulation, particularly the prefrontal cortex^11^. From a motivational perspective, mindfulness may promote stress reduction by satisfying basic psychological needs for autonomy, competence, and relatedness^12^.

A growing number of studies support the effectiveness of MBIs in reducing perceived stress across diverse populations. Randomised controlled trials and longitudinal research have shown significant decreases in self-reported stress levels among participants, regardless of whether interventions are delivered in person or digitally^13–16^. Systematic reviews and meta-analyses have further demonstrated the effectiveness of mindfulness-based interventions in reducing stress and improving mental health outcomes across diverse adult populations and delivery formats^17^^,18^. and hypertension, where MBIs have been associated with reductions in stress, anxiety, and depression. Promising evidence has also been reported for trauma-related conditions^19^ and smoking cessation, although results remain mixed^20^.

Recent randomised trials specifically highlight the effectiveness of MBIs in non-clinical adult populations when stress is measured with the PSS. For example, app-based mindfulness training has been shown to reduce perceived stress and enhance self-regulation among university students^21^. In contrast, short-term mindfulness training has been found to decrease stress in student samples^22^. These findings confirm that MBIs are adaptable across different delivery methods and effective in reducing stress as assessed by validated scales such as the PSS.

Despite this strong evidence base, gaps still exist. Previous evaluations often use varied stress measures, such as the Depression Anxiety Stress Scales (DASS) or composite tools, which limit comparisons across studies and obscure the specific effects of MBIs on subjective stress. To date, no systematic review has compiled evidence on MBIs for perceived stress measured solely with the PSS in non-clinical adult populations. Addressing this gap is essential for providing clinicians, researchers, and policymakers with reliable evidence to inform interventions for the general adult population.

The objective of this review was to conduct a systematic review and meta-analysis to evaluate the effectiveness of MBIs in reducing perceived stress, as measured by the PSS, in non-clinical adults.

## Methods

### Protocol and search strategies

The protocol, including the inclusion and exclusion criteria and analytical methods, was registered in PROSPERO on 4 August 2024 (CRD42024576505). The review followed PRISMA 2020 guidelines (see Supplementary Table 5). Systematic searches were conducted in Web of Science, PubMed/Medline, Scopus, PsycINFO, EBSCO, and Google Scholar for studies published between 1980 and 2025. The search period was anchored to 1980 to capture the earliest phase of formal research examining mindfulness and meditation-based interventions. The PubMed strategy combined MeSH and keyword terms and was restricted to English-language publications. PsycINFO searches used APA Thesaurus terms, while Scopus and Web of Science employed Title/Abstract/Keyword fields mapped to controlled vocabulary, and EBSCO relied on Subject Headings. Reference lists and citation tracking were reviewed for additional records, and Google Scholar was searched for studies that cited the original work. Full database-specific strategies are provided in Supplementary Table 1.

### Study selection

**Fig. 1:**
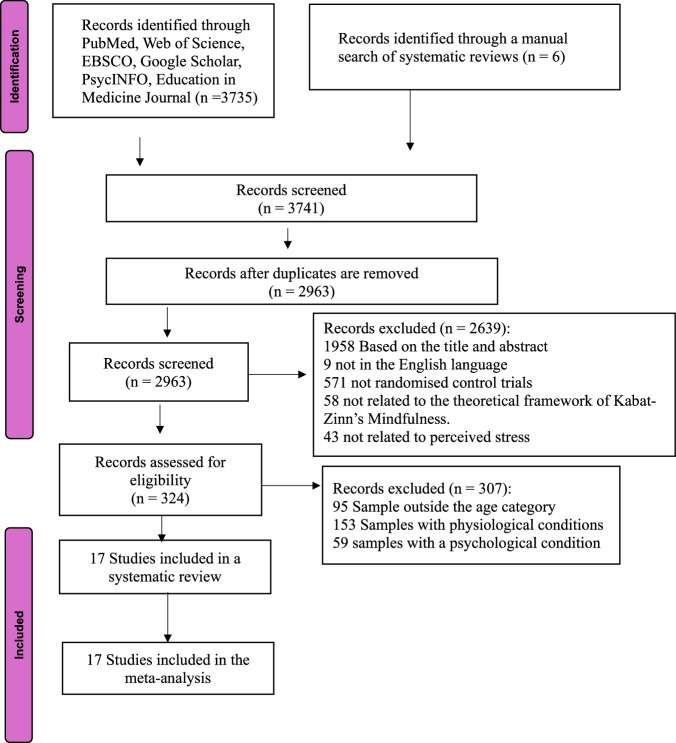
The PRISMA flow diagram of the study selection.

Study selection was performed independently by two reviewers, with discrepancies resolved through discussion or by the third reviewer. Studies were eligible if they employed randomised controlled trial designs, delivered mindfulness-based interventions grounded in Kabat-Zinn’s framework, included either active or inactive control groups, used validated outcome measures, specifically the Perceived Stress Scale (PSS), and reported both pre- and post-intervention PSS data. Studies were excluded if participants were under 18 years of age, involved clinical populations, received concurrent psychotherapy or meditation training outside the intervention, combined mindfulness with other psychoeducational therapies without a separate assessment, or if insufficient data were available. The study selection process is shown in the PRISMA flow diagram (see Fig. 1), and detailed criteria are summarised in Supplementary Table 2.

### Data extraction and risk of bias assessment

Data extraction and risk of bias assessment were conducted independently and blinded to author names, institutions, and publication details wherever possible. Extracted variables included author, year, population type, mean age, location, sample size, intervention type, delivery format, duration, and pre- and post-test PSS scores. Stress scores represented the mean perceived stress before and after the intervention in both the experimental and control groups, measured using the Perceived Stress Scale (PSS). When means or standard deviations were unavailable, study authors were contacted and missing values were estimated using RevMan 5.4 calculators. Methodological quality was assessed using the Jadad scale, which evaluates randomisation, blinding, and reporting of withdrawals. Studies scoring below four were considered low quality, and those scoring four or higher were classified as high quality. Details of the characteristics of included trials are presented in Table 1 and Supplementary Table 4. Study-level risk-of-bias assessments are provided in Supplementary Table 3.

**Table 1:** Characteristics of Included Studies

Study	Design	Population	*N*	Intervention	Comparator	Duration	Outcome	Risk of bias
Querstret et al.^41^	RCT	Working adults	118	Online MBCT	Waitlist	4 weeks	PSS (baseline, post)	Low–Moderate
Yang et al.^42^	RCT	Medical undergraduates	88	Headspace mindfulness meditation	Waitlist	30 days	PSS (baseline, post)	Moderate
Ireland et al.^43^	RCT	Intern doctors	44	MBSR group sessions	Extra break	10 weeks	PSS (baseline, post)	Moderate
Erogul et al.^44^	RCT	Medical students	58	MBSR weekly sessions	Waitlist	8 weeks	PSS (baseline, post)	Moderate
Xiong et al.^28^	RCT	College students	310	Digital MBSR	Waitlist	4 weeks	PSS (baseline, post)	Moderate
Min et al.^29^	RCT	Employees	63	Mindfulness training	Waitlist	4 weeks	PSS (baseline, post)	Moderate
Gallo et al.^30^	RCT	University students	136	MBRP group sessions	Waitlist	8 weeks	PSS (baseline, post)	Low–Moderate
Boden et al.^31^	RCT	Orthopaedic surgeons	24	Headspace app	Waitlist	8 weeks	PSS (baseline, post)	Moderate
Xu et al.^32^	RCT	ED staff	148	Headspace app	Waitlist	4 weeks	PSS (baseline, post)	Low–Moderate
Bartlett et al.^33^	RCT	Public sector employees	141	Smiling Mind program	Waitlist	8 weeks	PSS (baseline, post)	Moderate
Loh et al.^34^	RCT	Medical students	59	Brief mindfulness	Waitlist	4 weeks	PSS (baseline, post)	Moderate
Ritvo et al.^35^	RCT	University students	154	Virtual mindfulness community	Waitlist	8 weeks	PSS (baseline, post)	Low–Moderate
Sousa et al.^36^	RCT	University students	40	Brief mindfulness	Health activities	3 days	PSS (baseline, post)	High
Ameli et al.^37^	RCT	Healthcare professionals	78	Mindfulness self-care	Waitlist	5 weeks	PSS (baseline, post)	Moderate
Huberty et al.^38^	RCT	Undergraduates	88	Calm app	Waitlist	8 weeks	PSS (baseline, post)	Low–Moderate
Lin et al.^39^	RCT	Nurses	90	Modified MBSR	Waitlist	8 weeks	PSS (baseline, post)	Moderate
Champion et al.^40^	RCT	Healthy adults	62	Headspace meditation	Waitlist	30 days	PSS (baseline, post)	Moderate

*PSS* Perceived Stress Scale, *MBSR* Mindfulness-Based Stress Reduction, *MBCT* Mindfulness-Based Cognitive Therapy.

### Data analysis

Data analysis was conducted using Review Manager 5.3 (Cochrane Collaboration, Oxford, UK) to calculate effect sizes for between-group and within-group comparisons, expressed as standardised mean differences (SMDs) with 95% confidence intervals (CIs). A random-effects model was applied, and statistical significance was assessed with the Z-test (*p* < 0.05). Heterogeneity was evaluated using the *I* ^2^ statistic, with values greater than 50% considered substantial^20^.

Moderator analyses were conducted to explore potential sources of heterogeneity, including participant age, year of publication, geographic location, intervention duration, and mode of delivery. Subgroup analyses were used for categorical moderators such as geographic region and delivery format. At the same time, meta-regression was applied to continuous moderators only when data from at least ten trials were available, in line with standard methodological recommendations to ensure adequate statistical power and to reduce the risk of spurious associations in meta-regression analyses^21–23^. Subgroup analyses estimated between-group standardised mean differences (SMDs) with 95% confidence intervals (CIs) for post-intervention stress scores across North America, Europe, South America, Asia, and Oceania, as well as for direct versus indirect interventions. Statistical heterogeneity was assessed using the _I^−2^ statistic and Cochran’s Q test (*p*h).

Meta-regression models evaluated associations between pooled effect sizes and covariates, including publication year, mean age, sample size, and intervention duration, with coefficients, 95% CIs, and *p*-values reported. Sensitivity analyses assessed the robustness of the findings by sequentially excluding each study and recalculating the pooled effect sizes^24^. The Open Meta Analyst software was used for subgroup analysis, meta-regression, and sensitivity analysis^25^. Publication bias was evaluated with Egger’s regression test^26^, Begg and Mazumdar’s rank correlation test^27^, and visual inspection of funnel plots and standard error plots.

## Results

### Characteristics of Included Studies

A total of 3741 records were identified, of which 17 randomised controlled trials met the inclusion criteria (Table 1)^28–44^. Collectively, these trials enrolled 1641 participants, with 821 assigned to mindfulness-based intervention (MBI) groups and 820 to control groups. Publication years ranged from 2014 to 2025.

The populations studied were diverse, including university students^30,35,36,38^, medical students^34,44^, healthcare workers^32,37,43^, surgeons^31^, nurses^39^ and healthy adults^40^, with participant ages ranging from 18–65 years (mean range, 20–46 years).

Trials were conducted across North America^35,37,38,40,44^, South America^30,36^, Europe^41^, Asia^28,29,34,39^, and Oceania^32,33^, and tested interventions including mindfulness-based stress reduction (MBSR), modified MBSR, brief mindfulness training, smartphone- or app-based programmes (e.g., Headspace, Calm, Smiling Mind, online mindfulness-based cognitive therapy), and mindfulness-based self-care.

Nine trials used direct delivery formats (in-person or guided), whereas eight used digital platforms, with intervention durations ranging from 3 days to 10 weeks (median, 4–8 weeks).

Overall, methodological quality was high, with most studies scoring 6 and three studies scoring 8 on the Jadad scale. Additional study characteristics are provided in Table 1.

### Meta-analyses on baseline stress between the MBIs and the Control Groups

**Fig. 2:**
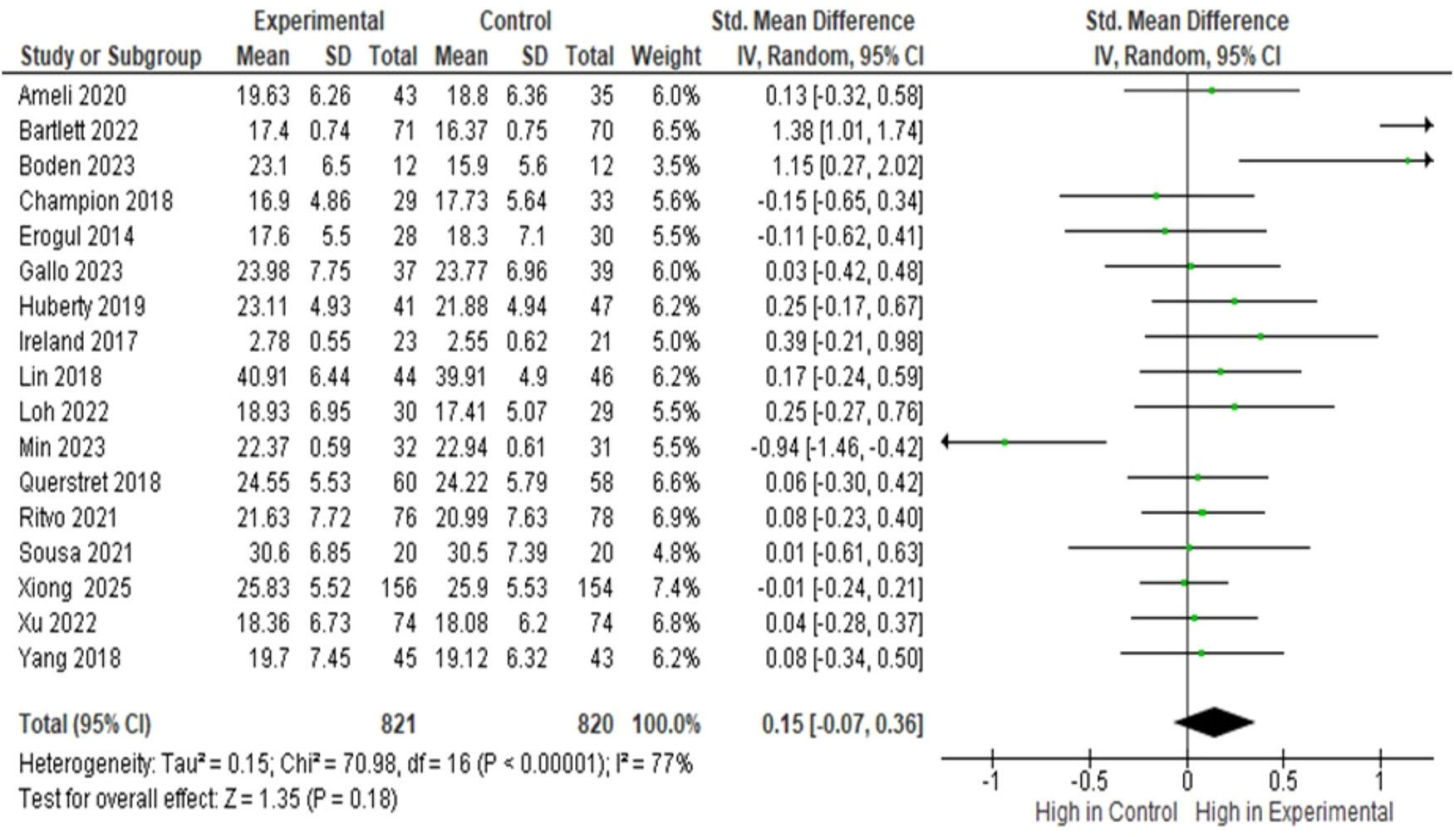
Forest Plot of Standardised Mean Difference in Baseline Perceived Stress Between the Experimental and Control Groups.

The effects of mindfulness-based interventions on perceived stress, as measured by the Perceived Stress Scale (PSS), were evaluated across 17 studies involving 1,641 participants (821 in the intervention group and 820 in the control group). The standardised mean difference (SMD) was used as the pooled effect size metric. At pre-intervention, perceived stress levels were comparable between the control and intervention groups (SMD = 0.150, 95% CI − 0.070 to 0.360, *p* = 0.180), indicating no baseline differences in stress. Substantial heterogeneity was observed (*I*^2^ = 77.0%, *p* < 0.0001) (Fig. 2).

### Meta-analyses on pre- vs. post-stress of the MBIs group

**Fig. 3:**
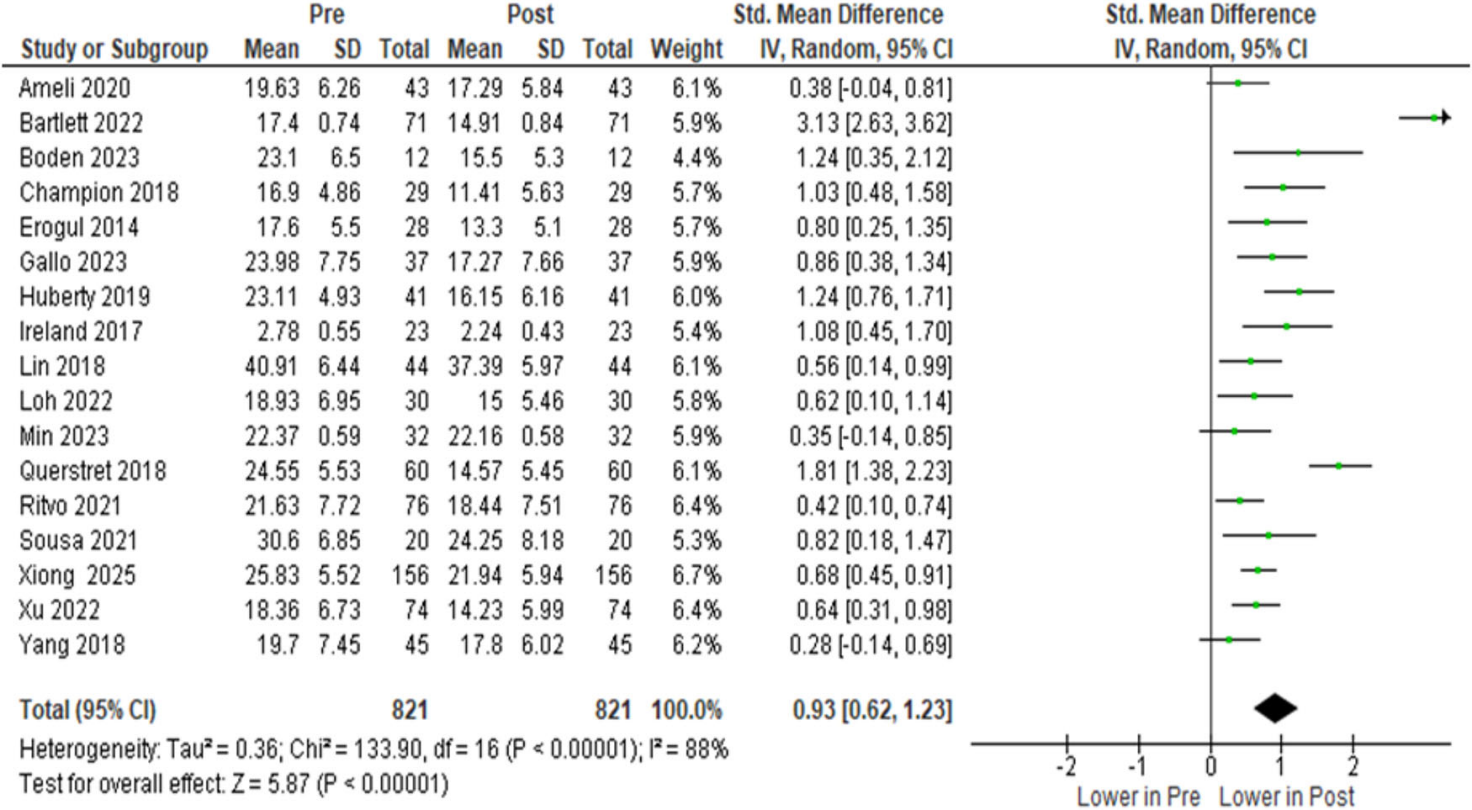
Forest plot of standardised mean difference in perceived stress between the pre- and post-condition of the experimental group.

The analysis of 17 studies involving 821 participants in the intervention group showed a significant reduction in perceived stress following mindfulness-based interventions. Despite substantial heterogeneity across studies (*I*² = 88.0%, *p* < 0.00001), the random-effects model indicated a marked post-intervention reduction in stress (SMD = 0.930, 95% CI 0.620 to 1.230, *p* < 0.00001) (Fig. 3).

### Meta-analyses on pre- vs. post-stress of the control group

**Fig. 4:**
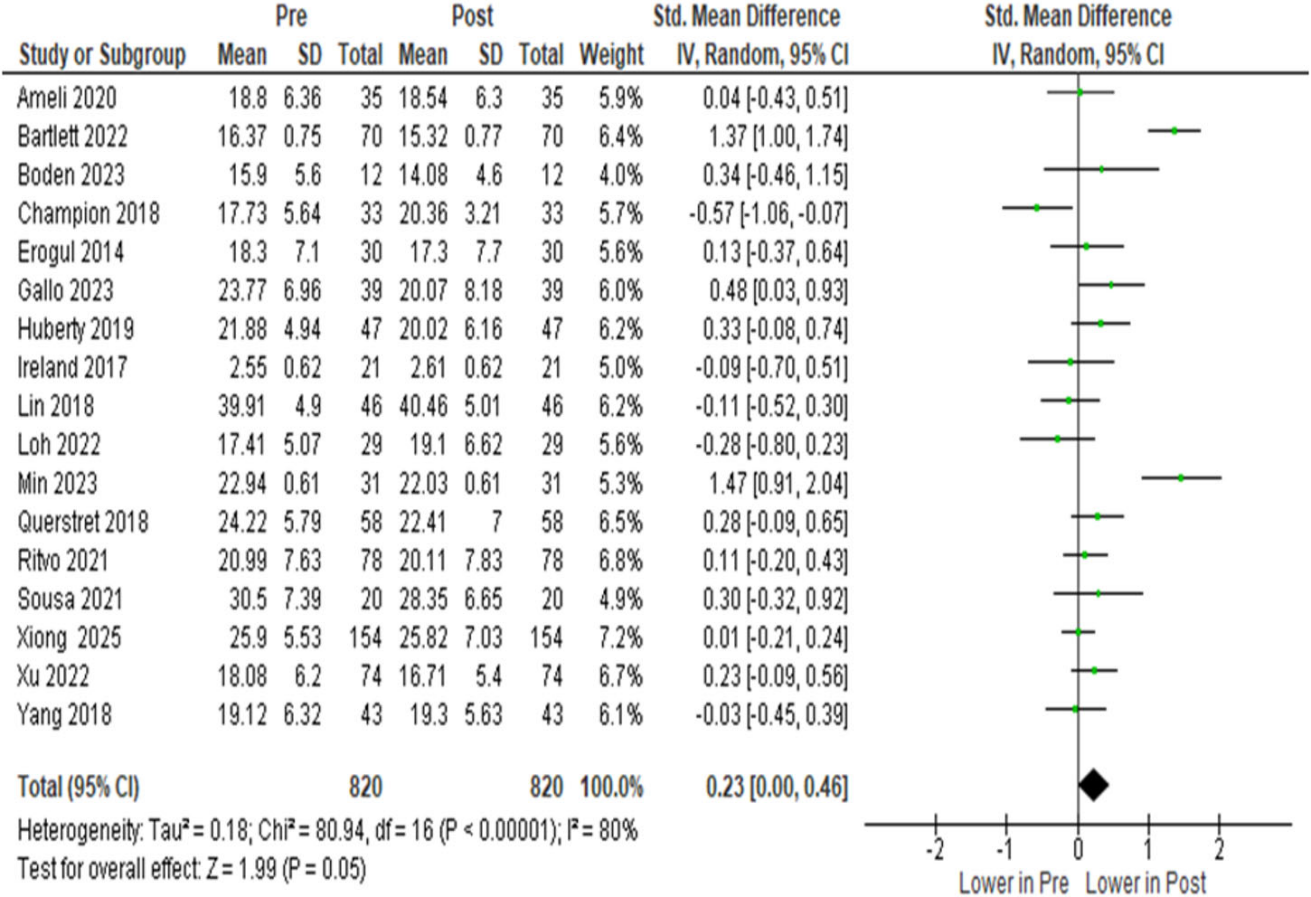
Forest plot of standardised mean difference in perceived stress between the pre- and post-condition of the control group.

The analysis of 17 studies involving 820 participants in the control group, which did not receive mindfulness-based interventions, showed a slight change in perceived stress from pre- to post-condition. Despite substantial heterogeneity across studies (*I*^2^ = 80.0%, *p* < 0.00001), the pooled effect size indicated a borderline significant change in stress (SMD = 0.230, 95% CI 0.000 to 0.460, *p* = 0.050). This finding suggests that perceived stress in control groups may change over time, potentially reflecting natural fluctuations or factors unrelated to mindfulness-based interventions (Fig. 4).

### Meta-analyses on post-intervention stress between the MBIs and the Control Groups

**Fig. 5:**
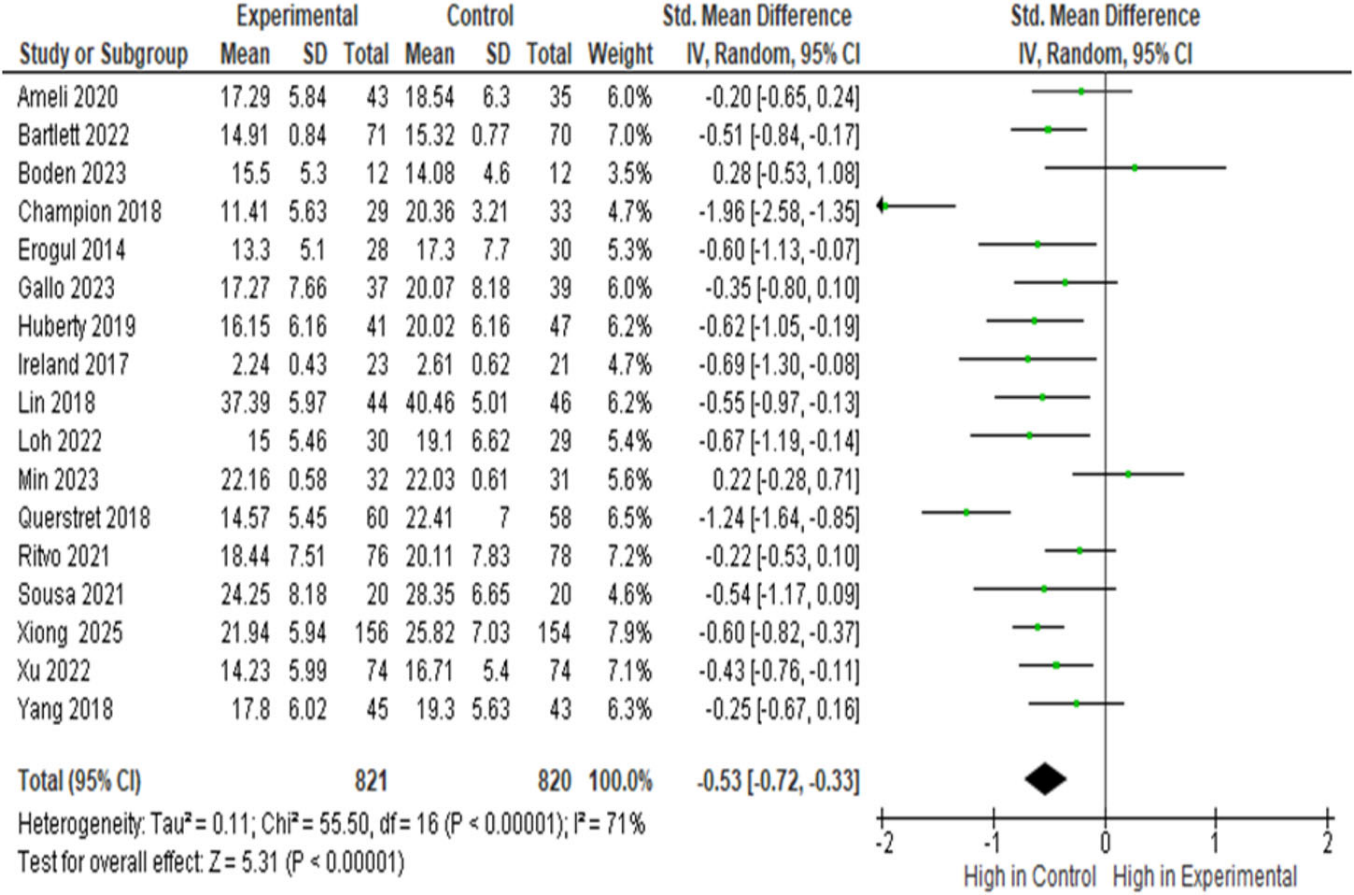
Forest plot of standardised mean difference in post perceived stress between the experimental and control groups.

Seventeen studies involving 1641 participants (821 in the intervention group and 820 in the control group) were included in the post-intervention stress level comparison. Post-intervention, perceived stress was higher in the control group than in the mindfulness-based intervention group (SMD = − 0.530, 95% CI − 0.720 to −0.330, *p* < 0.00001), indicating a significant reduction in stress associated with mindfulness-based interventions. Substantial heterogeneity was observed across studies (*I*² = 71.0%, *p* < 0.00001), reflecting variability in effect sizes (Fig. 5).

### Publication bias assessment

The assessment of publication bias yielded distinct findings for baseline and post-intervention perceived stress. For baseline perceived stress, the fail-safe *N* was 34 (*p* = 0.002), indicating moderate robustness. In contrast, Begg and Mazumdar’s rank correlation test (value = 0.118, *p* = 0.542) and Egger’s regression test (value = 0.539, *p* = 0.590) showed no evidence of publication bias, with the trim-and-fill method estimating zero missing studies. For post-intervention perceived stress, the fail-safe *N* was 594 (*p* < 0.001), indicating high robustness. Consistently, Begg and Mazumdar’s rank correlation test (value = − 0.029, *p* = 0.903) and Egger’s regression test (value = − 0.065, *p* = 0.948) were non-significant, and the trim-and-fill analysis again imputed zero missing studies. Visual inspection of the funnel plots showed a symmetrical distribution of effect sizes (Supplementary Figs. 1 and 2). Collectively, these findings suggest that publication bias is unlikely to have materially influenced the results, supporting the robustness of the observed effects (Table 2).

**Table 2:** Publication bias assessment of included randomised controlled trials

Test Name	Baseline Perceived Stress		Post Perceived Stress	
	value	*p*	value	*p*
Fail-Safe N	34	0.002	594	<.001
Begg and Mazumdar Rank Correlation	0.11765	0.542	−0.0294	0.903
Egger’s Regression	0.53933	0.59	−0.0652	0.948
Trim and Fill Number of Studies	0	.	0	.
Note. Fail-safe N Calculation Using the Rosenthal Approach				

Publication bias was examined using Rosenthal’s fail-safe N, Begg and Mazumdar rank correlation, and Egger’s regression test for both baseline and post-intervention perceived stress outcomes. No studies were imputed by the trim-and-fill method. p, probability value. Fail-safe N calculated using Rosenthal’s approach.

### Meta-regression

Meta-regression analyses showed no statistically significant associations between the pooled effect size and year of publication (coefficient = 0.569, *p* = 0.341), mean participant age (coefficient = −0.043, *p* = 0.827), sample size (coefficient = 0.020, *p* = 0.424), or intervention duration (coefficient = −0.391, *p* = 0.626). These results indicate that none of the examined study-level covariates significantly moderated the effects of mindfulness-based interventions on perceived stress (Supplementary Figs. 3–6).

### Subgroup analysis

#### Geographical area – baseline stress (MBIs vs. control groups)

Across regions, there were no statistically significant differences in baseline perceived stress between MBIs and control groups, indicating good baseline comparability. Effect sizes were small and non-significant in North America (SMD = 0.14, 95% CI: −0.04 to −0.32, *p* = 0.120, I² = 22.1%), Europe (SMD = −0.02, −0.31 to 0.28, *p* = 0.919, I² = 0%), South America (SMD = 0.02, −0.34 to −0.39, *p* = 0.902, I² = 0%), Oceania (SMD = −0.61, −0.20 to 1.41, *p* = 0.141, I² = 93%), and Asia (SMD = −0.12, −0.62 to 0.38, *p* = 0.644, I² = 78%).

#### Geographical area – within MBIs groups (baseline vs. post-intervention)

Significant reductions in perceived stress were observed following MBIs in all regions. Moderate effects were found in North America (SMD = 0.66, 0.33 to 0.99, *p* < 0.0001, I² = 64.1%) and Asia (SMD = 0.61, 0.43 to 0.79, *p* < 0.0001, I² = 0%), while large effects were noted in Europe (SMD = 1.44, 0.68 to 2.20, *p* = 0.0002, I² = 79.1%), South America (SMD = 0.85, 0.46 to 1.23, *p* < 0.0001, I² = 0%), and Oceania (SMD = 1.61, 0.11 to 3.12, *p* = 0.036, I² = 97.1%).

#### Geographical area – within control groups (baseline vs. post-intervention)

Control groups generally showed small and mostly non-significant changes in perceived stress. North America (SMD = 0.13, −0.05 to 0.31, *p* = 0.143, I² = 0%), Europe (SMD = −0.13, −0.96 to 0.70, *p* = 0.764, I² = 86.3%), Oceania (SMD = 0.52, −0.35 to 1.39, *p* = 0.240, I² = 92.5%), and Asia (SMD = 0.26, −0.51 to 1.02, *p* = 0.509, I² = 88.6%) did not show significant improvements, while South America showed a modest but significant reduction (SMD = 0.42, 0.05 to 0.78, *p* = 0.024, I² = 0%).

#### Geographical area – post-intervention stress (MBIs vs. control groups)

Significant post-intervention differences favoring MBIs were observed across all regions. Small to moderate effects were found in North America (SMD = −0.314, −0.511 to −0.116, *p* = 0.002, I² = 14.76%), South America (SMD = −0.414, −0.782 to −0.046, *p* = 0.028, I² = 0%), Oceania (SMD = −0.496, −0.715 to −0.278, *p* < 0.001, I² = 0%), and Asia (SMD = −0.424, −0.773 to −0.075, *p* = 0.017, I² = 67.19%), while a large effect was observed in Europe (SMD = −1.565, −2.264 to −0.865, *p* < 0.001, I² = 73.54%).

#### Medium of intervention – baseline stress (MBIs vs. control groups)

No significant baseline differences were observed for either delivery mode. Direct interventions showed an SMD of 0.01 ( − 0.22 to 0.24, *p* = 0.949, I² = 50.9%), while indirect interventions showed an SMD of 0.31 ( − 0.07 to 0.69, *p* = 0.109, I² = 86.2%).

#### Medium of intervention – within MBIs groups (baseline vs. post-intervention)

Both delivery modes resulted in significant reductions in perceived stress. Direct interventions showed a moderate effect (SMD = 0.59, 0.43 to 0.74, *p* < 0.0001, I² = 0%), whereas indirect interventions demonstrated a larger effect (SMD = 1.25, 0.62 to 1.87, *p* < 0.0001), although with substantial heterogeneity (I² = 93.6%).

#### Medium of intervention – within control groups (baseline vs. post-intervention)

Control groups did not show significant stress reductions for either delivery mode. Direct control interventions had an SMD of 0.22 ( − 0.10 to 0.54, *p* = 0.185, I² = 71.8%), and indirect control interventions had an SMD of 0.25 ( − 0.13 to 0.64, *p* = 0.201, I² = 86.6%).

#### Medium of intervention – post-intervention stress (MBIs vs. control groups)

Significant post-intervention differences favoring MBIs were observed for both delivery formats. Direct interventions showed a small to moderate effect (SMD = −0.368, −0.551 to −0.185, *p* < 0.001, I² = 25.86%), while indirect interventions demonstrated a larger effect (SMD = −0.676, −0.998 to −0.354, *p* < 0.001), with higher heterogeneity (I² = 81.49%).

These findings suggest that the interventions were effective across different regions and mediums, with varying magnitudes of effect (see Table 3).

**Table 3:** Subgroup analyses of mindfulness-based interventions on perceived stress

Subgroups		Number of Studies	SMD (95% CI)	*P*-Value	I^2^	P_h_
**Based on Area**	North America	6	−0.314 (−0.511, −0.116)	0.002	14.76%	0.320
	Europe	2	−1.565 (−2.264 -0.865)	< 0.001	73.54%	0.052
	South America	2	−0.414 (−0.782, −0.046)	0.028	0%	0.632
	Oceania	3	−0.496 (−0.715, −0.278)	< 0.001	0%	0.669
	Asia	4	−0.424 (−0.773 −0.075)	0.017	67.19%	0.027
**Medium of Intervention**	Direct	9	−0.368 (−0.551, −0.185)	< 0.001	25.86%	0.214
	Indirect	8	−0.676 (−0.998, −0.354)	< 0.001	81.49%	0.000

Subgroup analyses were performed by geographical area (North America, Europe, South America, Oceania, Asia) and medium of intervention (direct vs. indirect). Direct interventions were provided in either in-person or virtual guided formats, while indirect interventions involved self-guided digital platforms. Reported statistics include the number of studies, standardised mean difference (SMD) with 95% confidence intervals (CI), *p*-values, heterogeneity (I²), and heterogeneity test *p*-values (Ph).

### Sensitivity analysis

**Fig. 6:**
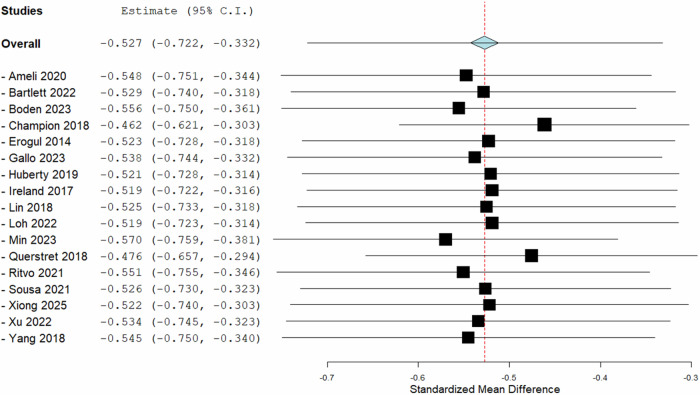
A forest plot of a leave-one-out meta-analysis of random effects represented the aggregate pooled effect size across 17 trials.

Sensitivity analyses indicated that the overall effect size remained stable when studies were sequentially excluded, supporting the robustness of the findings. The pooled standardised mean difference was −0.527 (95% CI − 0.722 to −0.332, *p* < 0.001). Exclusion of individual studies resulted in only minor changes to the pooled estimate, with the most significant deviations observed when excluding Champion (2018) (SMD = − 0.462) and Min (2023) (SMD = − 0.570). Overall, effect sizes ranged from −0.462 to −0.570, indicating that no single study exerted a disproportionate influence on the pooled effect (Fig. 6).

## Discussion

This systematic review and meta-analysis provide the first comprehensive synthesis of evidence on mindfulness-based interventions (MBIs) for reducing perceived stress in non-clinical adult populations. Across 17 randomised controlled trials involving 1,641 participants, MBIs were consistently associated with significant reductions in perceived stress compared with control conditions, supporting their effectiveness across diverse populations and delivery formats.

Baseline comparisons demonstrated no differences between intervention and control groups (SMD = 0.150, *p* = 0.180), indicating adequate randomisation and baseline comparability, consistent with previous workplace- and community-based mindfulness trials^11,12^. Within-group analyses showed substantial reductions in perceived stress among participants receiving MBIs (SMD = 0.930, *p* < 0.00001), in line with prior evidence linking mindfulness practice to improvements in emotional regulation and cognitive reappraisal through neural and psychological self-regulatory mechanisms^7,9^. By contrast, control groups exhibited only marginal improvements over time (SMD = 0.230, *p* = 0.050), likely reflecting natural fluctuations in stress, expectancy effects or regression to the mean, consistent with earlier findings in passive or waitlist-controlled trials^45,46^. Between-group post-intervention analyses further confirmed the superiority of MBIs (SMD = − 0.530, *p* < 0.00001), with effects consistently favouring intervention groups across studies.

Substantial heterogeneity was observed across baseline comparisons, within-group analyses for both MBIs and control groups, and post-intervention between-group comparisons, representing a key limitation of the evidence base. This heterogeneity is plausibly explained by pronounced variability in participant characteristics, baseline stress severity, intervention format and intensity, delivery mode, and control condition design across trials. The included studies enrolled highly heterogeneous non-clinical populations, ranging from college and medical students^28,34,35,38,42,44^ to healthcare professionals, including nurses, emergency department staff, intern doctors, and orthopaedic surgeons^31,32,37,39,43^, as well as working and public-sector employees^29,33,41^. These populations differ markedly in occupational demands, stress exposure, and coping resources, which was reflected in wide variation in baseline perceived stress levels, with reported PSS means ranging from approximately 16 to over 40^38,39^.

Intervention characteristics further contributed to variability. MBIs differed not only in delivery mode but also in content and intensity, spanning brief interventions lasting as little as 3 days^36^, app-based programmes of approximately 30 days^40,42^, and structured mindfulness-based stress reduction programmes lasting up to 10 weeks^43^. Differences in intervention “dose”, guidance intensity, and protocol standardisation are likely to yield differential magnitudes of stress reduction. Control conditions also varied, with most studies employing waitlist controls but others using active comparators such as self-care activities or additional rest breaks^29,36,43^, which may attenuate between-group effects and increase between-study dispersion. Although overall methodological quality was acceptable (Jadad scores 6–8), heterogeneity may also have been amplified by variation in assessor blinding, allocation concealment, and sample size, particularly in smaller trials involving specialised populations such as surgeons or intern doctors^31,43^.

To explore potential contributors to this heterogeneity, subgroup analyses by geographical region and medium of intervention delivery, followed by meta-regression, were also conducted. Baseline subgroup analyses across continents revealed no statistically significant differences in perceived stress between MBI and control groups, supporting adequate randomisation across regions. Nevertheless, baseline heterogeneity remained high in Oceania and Asia, plausibly reflecting the inclusion of heterogeneous populations within these regions. For example, studies conducted in Oceania included emergency department staff, intern doctors, and public-sector employees^32,33,43^. In contrast, Asian studies encompassed college students, medical students, nurses, and employees^28,29,34,39^, representing diverse academic and healthcare contexts with distinct stress determinants.

Within-group analyses of MBIs demonstrated significant reductions in perceived stress across all regions, although effect sizes and heterogeneity varied. Larger effects and higher heterogeneity observed in Europe and Oceania may reflect greater variability in intervention intensity and occupational context, including longer or more intensive programmes such as 10-week MBSR interventions^43^ and 8-week workplace-based mindfulness programmes^33^. In contrast, Asian studies showed relatively more homogeneous effects, potentially due to greater consistency in intervention duration (predominantly 4–8 weeks) and the predominance of student or healthcare populations exposed to broadly similar academic or clinical stressors^28,34,39^.

Delivery-mode subgroup analyses further clarified sources of variability. No baseline differences were observed between intervention and control groups for either direct or indirect delivery formats, supporting baseline equivalence. However, heterogeneity was higher among indirectly delivered interventions, plausibly reflecting the wide range of digital platforms used, including Headspace, Calm, Smiling Mind, and virtual mindfulness communities, which differ substantially in content structure, guidance, and user engagement^31–33,35,38^. Within-group analyses indicated that both delivery modes were effective in reducing perceived stress. However, indirect interventions demonstrated larger pooled effects accompanied by greater heterogeneity, likely driven by differences in programme duration, adherence, and self-guided engagement^32,40,41^. By comparison, direct interventions such as MBSR, which follow more standardised protocols, were associated with comparatively lower heterogeneity.

Meta-regression analyses examining publication year, sample size, mean age, and intervention duration did not identify any statistically significant moderators of effect size. These null findings suggest that heterogeneity cannot be attributed to a single linear study-level characteristic alone and is more likely driven by complex interactions among population type, intervention format, baseline stress severity, and control condition design.

Assessment of publication bias supported the robustness of the findings, with moderate fail-safe *N* values for baseline outcomes and substantially larger values for post-intervention outcomes, alongside non-significant Begg’s rank correlation and Egger’s regression tests^26,27^. Funnel plots and trim-and-fill analyses indicated a low risk of bias, consistent with established methodological standards in psychological meta-analysis^47,48^. Sensitivity analyses further demonstrated that no single study disproportionately influenced the pooled effect, with stable estimates across leave-one-out analyses^24,25^.

Despite these strengths, substantial heterogeneity remains a central limitation. Variation in populations, intervention dose, delivery channels, and control conditions complicates interpretation of pooled effect sizes and represents a persistent challenge in synthesising mindfulness-based interventions^20,23^. Moreover, the focus on immediate post-intervention outcomes limits conclusions regarding long-term sustainability, and incomplete blinding and inconsistent reporting of randomisation procedures reduce confidence in some individual trials. Additional contextual factors, including cultural influences, instructor expertise, and participant adherence, may further influence outcomes and restrict generalisability.

Taken together, the findings provide strong evidence that MBIs significantly lower perceived stress in non-clinical adult populations across various delivery formats and cultural settings. While heterogeneity remains considerable, it reflects meaningful clinical and methodological differences rather than inconsistency in the direction of effect. These results endorse MBIs as practical, scalable, and effective methods for reducing stress, while highlighting the need for future research to use more standardised comparators, optimise intervention dosing, clarify effects related to delivery channels, and evaluate longer-term outcomes to enhance comparability and implementation.

## Supplementary information

Supplementary information

## Data Availability

While most of the data generated during this study are included in this article and supplementary table, additional data are available from the corresponding author upon request.
