# Supplementary material for: Effects of mindfulness-based interventions on perceived stress among non-clinical adults: a systematic review and meta-analysis
Source: Npj Ment Health Res. 2026 Feb 3;5:9. doi: 10.1038/s44184-026-00188-4 (PMC12868674; doi:10.1038/s44184-026-00188-4)
Supplement: Supplementary file 1 — Supplementary information [file 44184_2026_188_MOESM1_ESM.pdf]

Effects of Mindfulness-Based Interventions on Perceived Stress among Non-Clinical Adults: A Systematic Review and Meta-Analysis

Supplementary Information

This file contains:

- Supplementary Table 1. Database-specific search strategies
- Supplementary Table 2. Inclusion and exclusion criteria
- Supplementary Table 3. Risk of bias summary (Jadad sub-items, revised)
- Supplementary Table 4. Raw dataset of included randomised controlled trials
- Supplementary Table 5. PRISMA 2020 Checklist (27 items)
- Supplementary Figure 1. Funnel plot asymmetry for Egger's regression test on baseline perceived stress
- Supplementary Figure 2. Funnel plot asymmetry for Egger's regression test on post perceived stress
- Supplementary Figure 3. Meta-regression analysis based on year of publication
- Supplementary Figure 4. Meta-regression analysis based on the mean age of participants
- Supplementary Figure 5. Meta-regression analysis based on sample size
- Supplementary Figure 6. Meta-regression analysis based on Intervention duration

Supplementary Table 1. Database-specific search strategies

Database	Controlled vocabulary / MeSH terms	Intervention-related terms	Outcome-related terms	Population-related terms	Example combined string
PubMed/Medline	“mindfulness”[MeSH]; “stress, psychological”[MeSH]; “Perceived Stress Scale”[Supplementary Concept]	Mindfulness-Based Stress Reduction; Mindfulness-Based Interventions	perceived stress; PSS; psychological well-being; stress reduction	adults; college students; employees	(“mindfulness”[MeSH] OR “mindfulness”[All Fields]) AND (“stress, psychological”[MeSH] OR “psychological stress”[All Fields] OR “perceived stress” OR “Perceived Stress Scale”[Supplementary Concept])
PsycINFO	APA Thesaurus: Mindfulness; Stress, Psychological; Perceived Stress Scale	same as above	same as above	same as above	(DE “Mindfulness”) AND (DE “Stress, Psychological” OR “Perceived Stress Scale”)
Scopus	Title/Abstract/Keywords: mindfulness; psychological stress; PSS	same as above	same as above	same as above	(TITLE-ABS-KEY(“mindfulness”) AND TITLE-ABS-KEY(“psychological stress” OR “perceived stress” OR “perceived stress scale”))
Web of Science	Keyword Plus / Topic: mindfulness; psychological stress; PSS; stress reduction	same as above	same as above	same as above	TS=(mindfulness AND (“psychological stress” OR “perceived stress” OR “perceived stress scale”))
EBSCO (CINAHL)	CINAHL Subject Headings: Mindfulness; Stress, Psychological; PSS	same as above	same as above	same as above	(MH “Mindfulness”) AND (MH “Stress, Psychological” OR “Perceived Stress Scale”)
Google Scholar	Keyword search only	same as above	same as above	same as above	“mindfulness” AND “perceived stress” AND “Perceived Stress Scale”

Note: PSS = Perceived Stress Scale; MH = Medical Subject Heading; DE = Descriptor.

Supplementary Table 2. Inclusion and exclusion criteria

Inclusion criteria	Exclusion criteria
Experimental groups received mindfulness-based interventions (with or without guidance), based on Kabat-Zinn's theoretical framework of mindfulness.	Participants under 18 years old.
Randomised controlled trial design.	Studies involving participants with diagnosed physical or mental health conditions.
Control groups were either active or inactive.	Participants receiving concurrent psychotherapy, counselling, or meditation training outside the study intervention.
Validated outcome measures (specifically the Perceived Stress Scale, PSS) were used to assess the effect of mindfulness on stress.	Studies combining mindfulness with other psychoeducational therapies, unless the effects of mindfulness can be assessed separately.
Pre- and post-intervention data on PSS are available.	Studies with insufficient data for analysis.

Note: PSS = Perceived Stress Scale.

Supplementary Table 3. Risk of bias summary (Jadad sub-items, revised)

Citation (Year)	Randomization reported	Randomization method appropriate	Assessor blinding	Participant blinding	Blinding method appropriate	Withdrawals/dropouts described	Allocation concealment	Other (e.g. prereg/ITT)	Jadad total
Xiong et al., 2025	Yes	Yes	No	No	No	Yes	NR	Trial preregistered	6
Min et al., 2023	Yes	Yes	No	No	No	Yes	NR	3-arm RCT, prespecified	6
Gallo et al., 2023	Yes	Yes	No	No	No	Yes	NR	–	6
Boden et al., 2023	Yes	Yes	No	No	No	Yes	NR	–	6
Xu et al., 2022	Yes	Yes	No	No	No	Yes	NR	–	6
Bartlett et al., 2022	Yes	Yes	No	No	No	Yes	NR	Protocol published	6
Loh et al., 2022	Yes	Yes	No	No	No	Yes	NR	–	6
Ritvo et al., 2021	Yes	Yes	No	No	No	Yes	NR	Outcomes prespecified	6
Sousa et al., 2021	Yes	Yes	No	No	No	Yes	NR	–	6
Ameli et al., 2020	Yes	Yes	No	No	No	Yes	NR	–	6
Huberty et al., 2019	Yes	Yes	Yes	No	Yes	Yes	Yes	–	8
Lin et al., 2018	Yes	Yes	No	No	No	Yes	NR	–	6
Champion et al., 2018	Yes	Yes	No	No	No	Yes	NR	–	6

Querstret et al., 2018	Yes	Yes	Yes	No	Yes	Yes	Yes	–	8
Yang et al., 2018	Yes	Yes	No	No	No	Yes	NR	–	6
Ireland et al., 2017	Yes	Yes	No	No	No	Yes	NR	–	6
Erogul et al., 2014	Yes	Yes	Yes	No	Yes	Yes	Yes	–	8

Yes = reported/appropriate; No = not reported/inappropriate; NR = not reported. Notes: Participant blinding is generally not feasible in mindfulness interventions. Assessor blinding was sometimes used in higher-quality studies. Totals correspond to extracted Jadad scores (0–8).

Supplementary Table 4. Raw Dataset of included randomised controlled trials

Citation (First author, Year)	DOI	Setting	Population (mean age)	N total / Exp / Ctrl	Intervention & delivery	PSS Exp Pre (Mean±SD)	PSS Exp Post (Mean±SD)	PSS Ctrl Pre (Mean±SD)	PSS Ctrl Post (Mean±SD)
Xiong et al., 2025[1]	10.3389/fpsyt.2025.1644370	University	College students (19.26)	310 / 156 / 154	MBSR – digital mindfulness (Indirect)	25.83±5.52	21.94±5.94	25.90±5.53	25.82±7.03
Min et al., 2023[2]	10.2196/42851	Workplace	Employees (38.65)	63 / 32 / 31	Mindfulness-based intervention (Direct)	22.37±0.59	22.16±0.58	22.94±0.61	22.03±0.61
Gallo et al., 2023[3]	10.1186/s13033-023-00604-8	University	University students (24)	136 / 37 / 39	Mindfulness-Based Relapse Prevention (Direct)	23.98±7.75	17.27±7.66	23.77±6.96	22.07±8.18

Boden et al., 2023[4]	10.2106/JBJS.OA.22.00114	Residency program	Orthopaedic resident surgeons (30.6)	24 / 12 / 12	Headspace app (Indirect)	23.10±6.50	15.50±5.30	15.90±5.60	14.80±4.60
Xu et al., 2022[5]	10.1111/1742-6723.13836	Hospital (ED staff)	Emergency Dept staff (40)	148 / 74 / 74	Headspace app (Indirect)	18.36±6.73	14.23±5.99	18.08±6.20	16.71±5.40
Bartlett et al., 2022[6]	10.2196/40754	Government workplace	State Service employees (49)	141 / 71 / 70	Smiling Mind Workplace Program (Indirect)	17.40±0.74	14.91±0.84	16.37±0.75	15.32±0.77
Loh et al., 2022[7]	10.21315/eimj2022.14.2.1	University	Medical students (20.47)	59 / 30 / 29	Brief mindfulness-based intervention (Direct)	18.93±6.95	15.00±5.46	17.41±5.07	19.10±6.62
Ritvo et al., 2021[8]	10.2196/23491	University	University students (23.1)	154 / 76 / 78	Mindfulness Virtual Community (Direct)	21.63±7.72	18.44±7.51	20.99±7.63	20.11±7.83
Sousa et al., 2021[9]	10.1186/s40359-021-00520-x	University	University students (24.15)	40 / 20 / 20	Brief mindfulness-based training (Direct)	30.60±6.85	24.25±8.18	30.50±7.39	28.35±6.65
Ameli et al., 2020[10]	10.1001/jamanetworkopen.2020.13424	Healthcare	Health care professionals (32)	78 / 43 / 35	Mindfulness-based self-care (Direct)	19.63±6.26	17.29±5.84	18.80±6.36	18.54±6.30

Huberty et al., 2019[11]	10.2196/14273	University	Undergraduates (20.41)	88 / 41 / 47	Calm app (Indirect)	23.11±4.93	16.15±6.16	21.88±4.94	20.02±6.16
Lin et al., 2018[12]	10.1177/2165079918801633	Healthcare	Nurses (32.86)	90 / 44 / 46	Modified MBSR (Direct)	40.91±6.44	37.39±5.97	39.91±4.90	40.76±5.01
Champion et al., 2018[13]	10.1371/journal.pone.0209482	Community	Healthy adults (39.13)	62 / 29 / 33	Headspace app (Indirect)	16.90±4.86	11.41±5.63	17.73±5.64	20.36±3.21
Querstret et al., 2018[14]	10.1007/s12671-018-0925-0	Workplace	Working population (40.68)	118 / 60 / 58	Online MBCT (Indirect)	24.55±5.53	14.57±5.45	24.22±5.79	22.41±7.00
Yang et al., 2018[15]	10.1089/acm.2015.0301	University	Medical undergraduates (25.11)	88 / 45 / 43	Headspace app (Indirect)	19.70±7.45	17.08±6.02	19.12±6.32	19.30±5.63
Ireland et al., 2017[16]	10.1080/0142159X.2017.1294749	Hospital (ED)	Intern doctors (26.88)	44 / 23 / 21	MBSR (Direct)	2.78±0.55	2.42±0.43	2.55±0.62	2.61±0.62
Erogul et al., 2014[17]	10.1080/10401334.2014.945025	University	1st-year medical students (23.5)	58 / 28 / 30	MBSR (Direct)	17.60±5.50	13.30±5.10	18.30±7.10	17.30±7.70

NR = not reported; PSS = Perceived Stress Scale; Exp = Experimental; Ctrl = Control. Funding: This research received no external funding. Competing interests: The authors declare that they have no competing interests.

PRISMA

Supplementary Table 5. PRISMA 2020 Checklist

Section and Topic	Item #	Checklist item	Location where item is reported
**TITLE**			
Title	1	Identify the report as a systematic review.	Page 1
**ABSTRACT**			
Abstract	2	See the PRISMA 2020 for Abstracts checklist.	Page 2
**INTRODUCTION**			
Rationale	3	Describe the rationale for the review in the context of existing knowledge.	Page 2-4
Objectives	4	Provide an explicit statement of the objective(s) or question(s) the review addresses.	Page 4
**METHODS**			
Eligibility criteria	5	Specify the inclusion and exclusion criteria for the review and how studies were grouped for the syntheses.	Supplementary Table 2
Information sources	6	Specify all databases, registers, websites, organisations, reference lists and other sources searched or consulted to identify studies. Specify the date when each source was last searched or consulted.	Supplementary Table 1
Search strategy	7	Present the full search strategies for all databases, registers and websites, including any filters and limits used.	Page 4
Selection process	8	Specify the methods used to decide whether a study met the inclusion criteria of the review, including how many reviewers screened each record and each report retrieved, whether they worked independently, and if applicable, details of automation tools used in the process.	Page 5
Data collection process	9	Specify the methods used to collect data from reports, including how many reviewers collected data from each report, whether they worked independently, any processes for obtaining or confirming data from study investigators, and if applicable, details of automation tools used in the process.	Page 5-6
Data items	10a	List and define all outcomes for which data were sought. Specify whether all results that were compatible with each outcome domain in each study were sought (e.g. for all measures, time points, analyses), and if not, the methods used to decide which results to collect.	Page 5-6
	10b	List and define all other variables for which data were sought (e.g. participant and intervention characteristics, funding sources). Describe any assumptions made about any missing or unclear information.	Table 1
Study risk of bias assessment	11	Specify the methods used to assess risk of bias in the included studies, including details of the tool(s) used, how many reviewers assessed each study and whether they worked independently, and if applicable, details of automation tools used in the process.	Page 5-6, Supplementary Table 3
Effect measures	12	Specify for each outcome the effect measure(s) (e.g. risk ratio, mean difference) used in the synthesis or presentation of results.	Page 6
Synthesis methods	13a	Describe the processes used to decide which studies were eligible for each synthesis (e.g. tabulating the study intervention characteristics and comparing against the planned groups for each synthesis (item #5)).	
	13b	Describe any methods required to prepare the data for presentation or synthesis, such as handling of missing summary statistics, or data conversions.	Page 6
	13c	Describe any methods used to tabulate or visually display results of individual studies and syntheses.	Page 6
	13d	Describe any methods used to synthesize results and provide a rationale for the choice(s). If meta-analysis was performed, describe the model(s), method(s) to identify the presence and extent of statistical heterogeneity, and software package(s) used.	Page 6
	13e	Describe any methods used to explore possible causes of heterogeneity among study results (e.g. subgroup analysis, meta-regression).	Page 6
	13f	Describe any sensitivity analyses conducted to assess robustness of the synthesized results.	Page 6
Reporting bias assessment	14	Describe any methods used to assess risk of bias due to missing results in a synthesis (arising from reporting biases).	
Certainty assessment	15	Describe any methods used to assess certainty (or confidence) in the body of evidence for an outcome.	NA
**RESULTS**			
Study selection	16a	Describe the results of the search and selection process, from the number of records identified in the search to the number of studies included in the review, ideally using a flow diagram.	Figure 1
	16b	Cite studies that might appear to meet the inclusion criteria, but which were excluded, and explain why they were excluded.	Figure 1

PRISMA

Section and Topic	Item #	Checklist item	Location where item is reported
Study characteristics	17	Cite each included study and present its characteristics.	Table 1
Risk of bias in studies	18	Present assessments of risk of bias for each included study.	Page 8-9
Results of individual studies	19	For all outcomes, present, for each study: (a) summary statistics for each group (where appropriate) and (b) an effect estimate and its precision (e.g. confidence/credible interval), ideally using structured tables or plots.	Page 7,8, Figure 2-5
Results of syntheses	20a	For each synthesis, briefly summarise the characteristics and risk of bias among contributing studies.	Page 7, Table 1
	20b	Present results of all statistical syntheses conducted. If meta-analysis was done, present for each the summary estimate and its precision (e.g. confidence/credible interval) and measures of statistical heterogeneity. If comparing groups, describe the direction of the effect.	Figure 2-5
	20c	Present results of all investigations of possible causes of heterogeneity among study results.	Page 9-10
	20d	Present results of all sensitivity analyses conducted to assess the robustness of the synthesized results.	Page 10, Figure 6
Reporting biases	21	Present assessments of risk of bias due to missing results (arising from reporting biases) for each synthesis assessed.	Page 8-9
Certainty of evidence	22	Present assessments of certainty (or confidence) in the body of evidence for each outcome assessed.	Figure 2-5
**DISCUSSION**			
Discussion	23a	Provide a general interpretation of the results in the context of other evidence.	Page 10-12
	23b	Discuss any limitations of the evidence included in the review.	Page 12
	23c	Discuss any limitations of the review processes used.	Page 12
	23d	Discuss implications of the results for practice, policy, and future research.	Page 12-13
**OTHER INFORMATION**			
Registration and protocol	24a	Provide registration information for the review, including register name and registration number, or state that the review was not registered.	Prospero (CRD42024576505)
	24b	Indicate where the review protocol can be accessed, or state that a protocol was not prepared.	CRD42024576505
	24c	Describe and explain any amendments to information provided at registration or in the protocol.	NA
Support	25	Describe sources of financial or non-financial support for the review, and the role of the funders or sponsors in the review.	Page 13
Competing interests	26	Declare any competing interests of review authors.	Page 13
Availability of data, code and other materials	27	Report which of the following are publicly available and where they can be found: template data collection forms; data extracted from included studies; data used for all analyses; analytic code; any other materials used in the review.	Supplementary Table 4

From: Page MJ, McKenzie JE, Bossuyt PM, Boutron I, Hoffmann TC, Mulrow CD, et al. The PRISMA 2020 statement: an updated guideline for reporting systematic reviews. BMJ 2021;372:n71. doi: 10.1136/bmj.n71. This work is licensed under CC BY 4.0. To view a copy of this license, visit https://creativecommons.org/licenses/by/4.0/

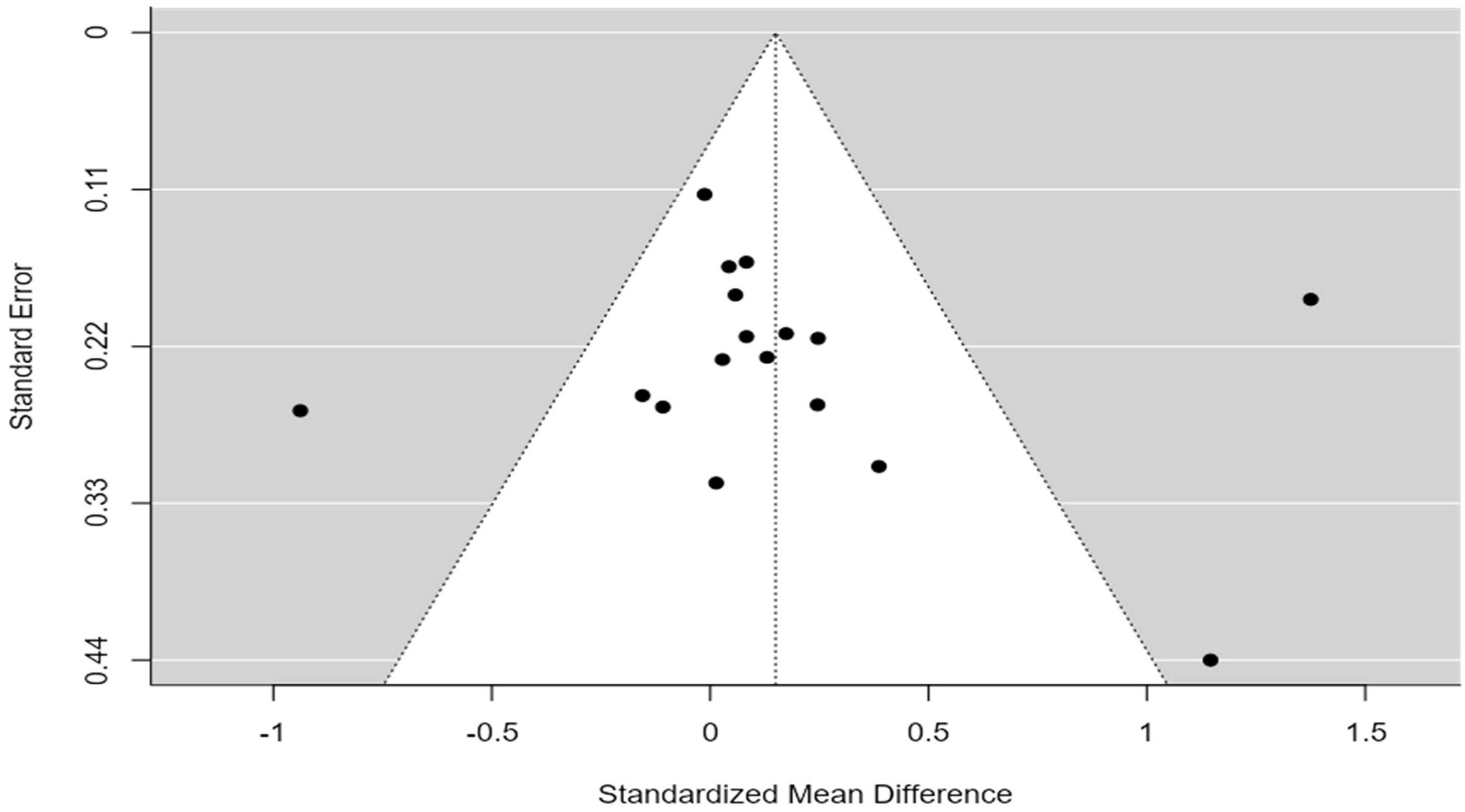

Supplementary Figure 1. Funnel plot asymmetry for Egger's regression test on baseline perceived stress

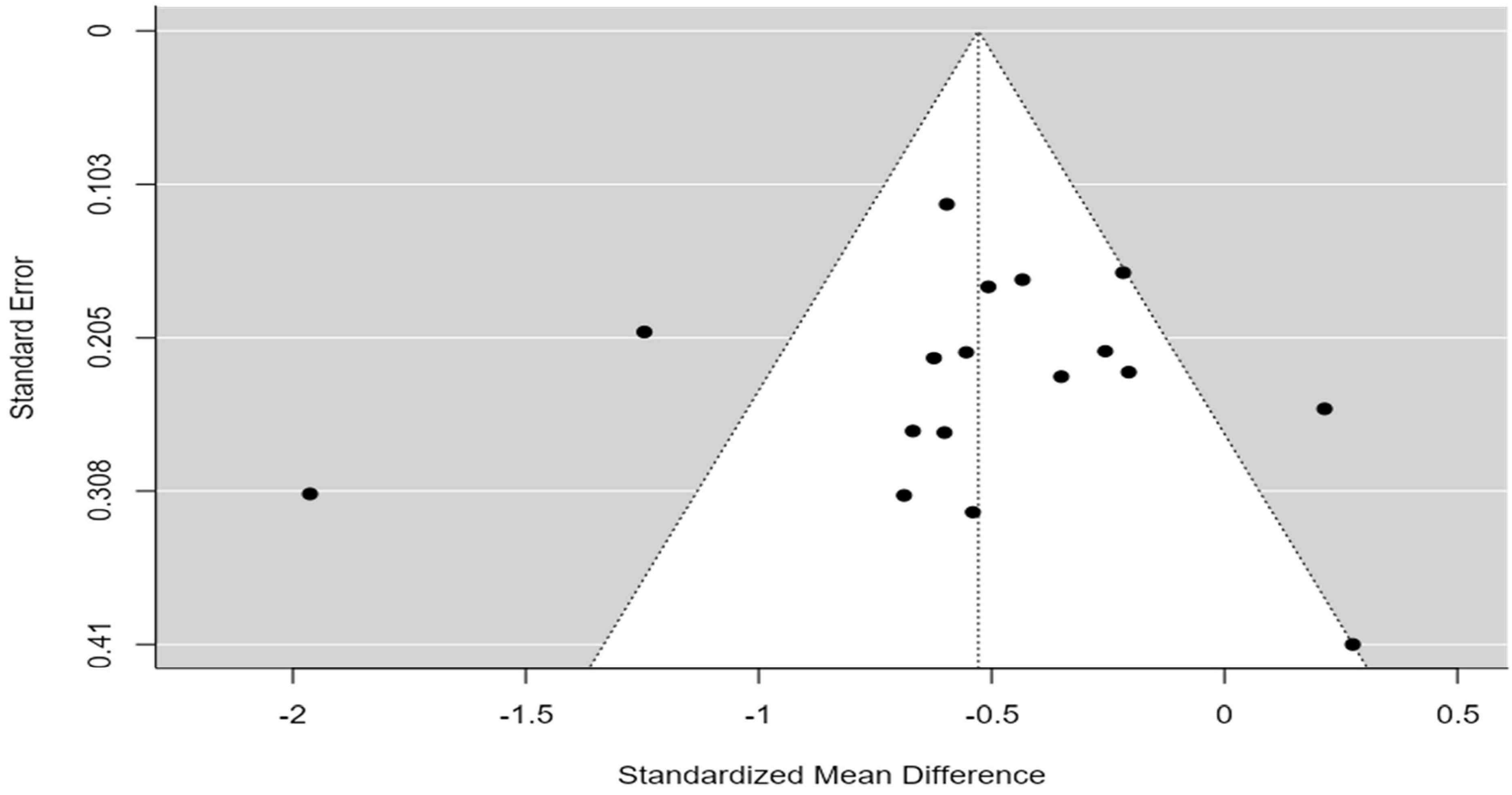

Supplementary Figure 2. Funnel plot asymmetry for Egger's regression test on post perceived stress

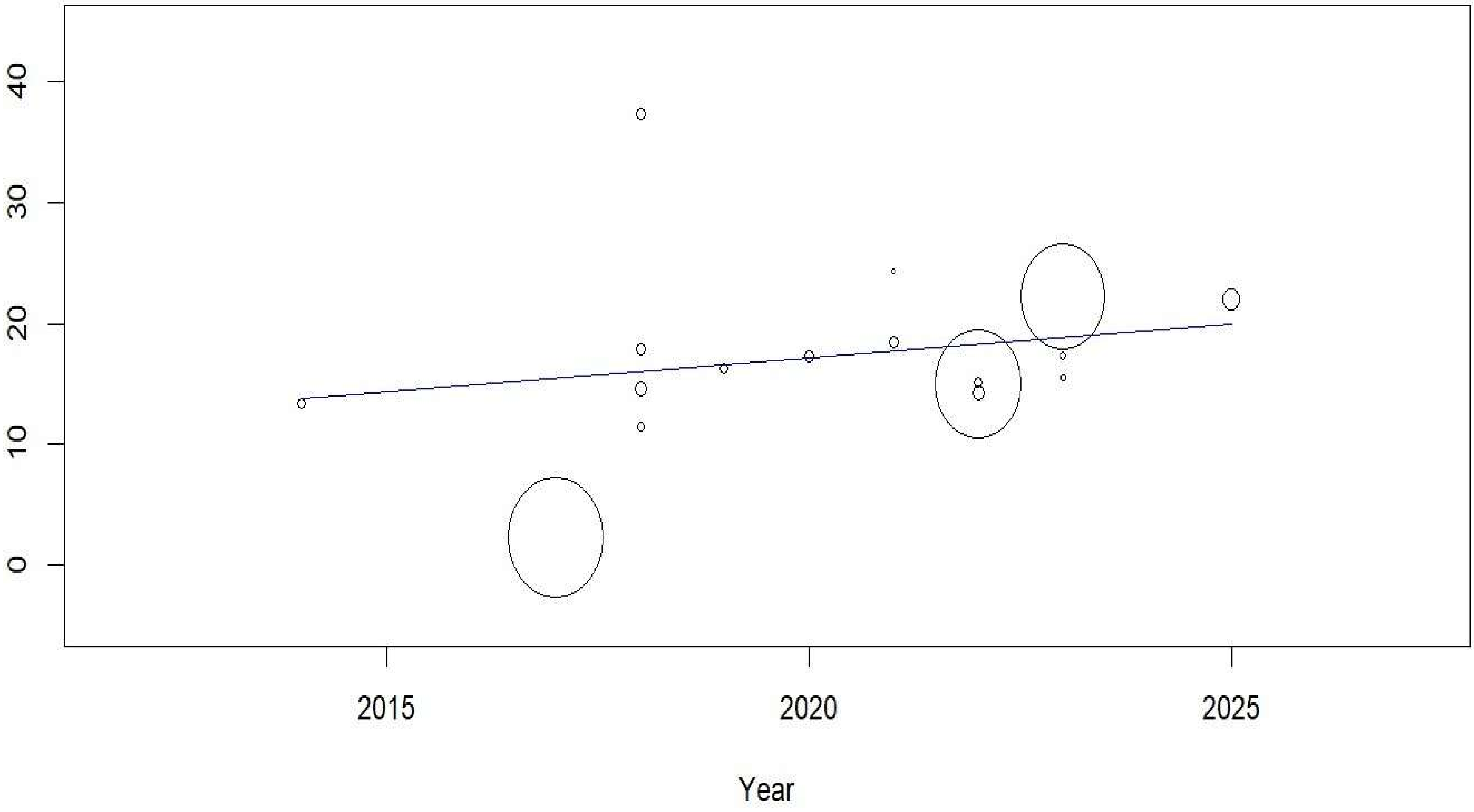

Supplementary Figure 3. Meta-regression analysis based on year of publication

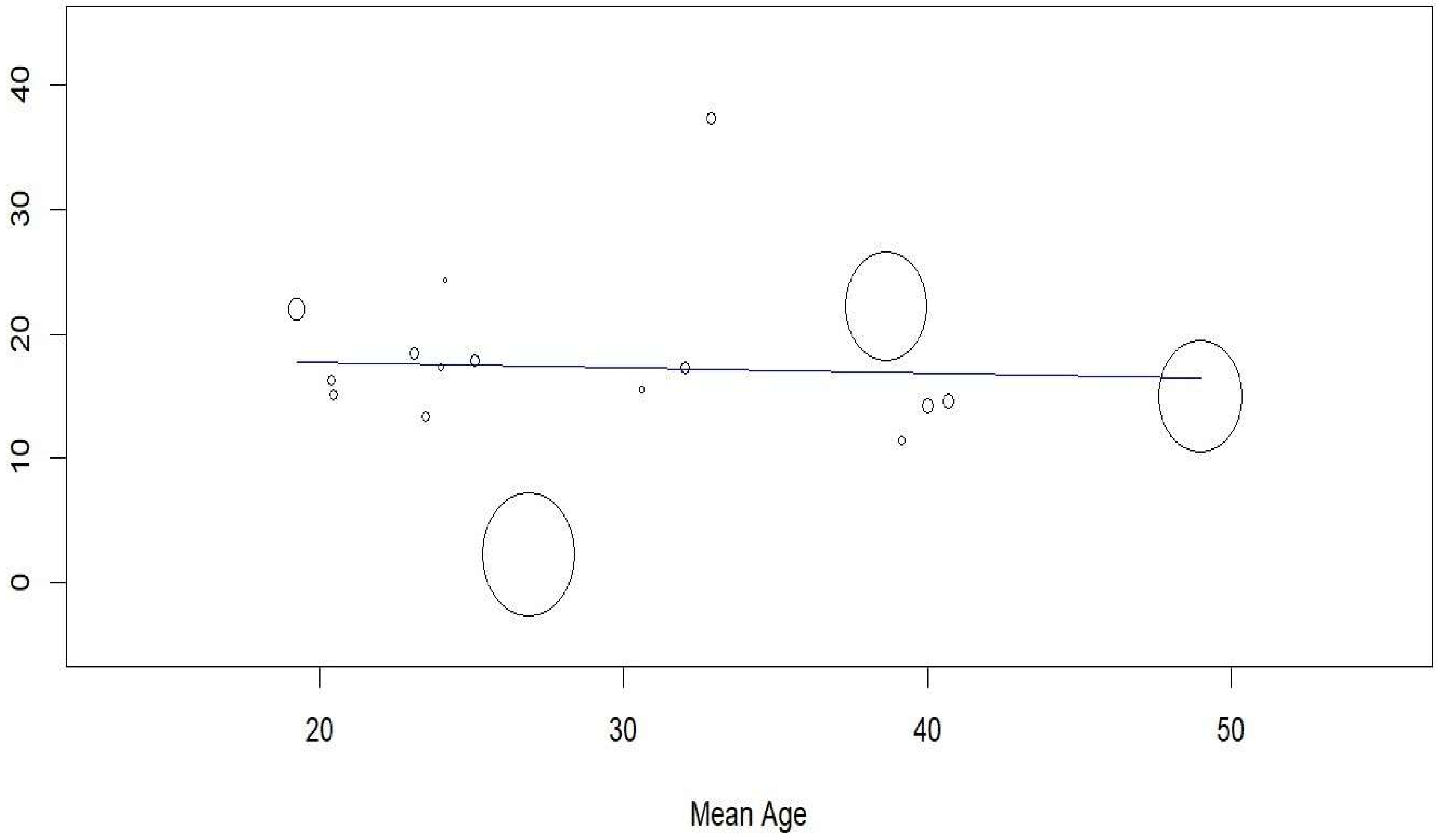

Supplementary Figure 4. Meta-regression analysis based on the mean age of participants

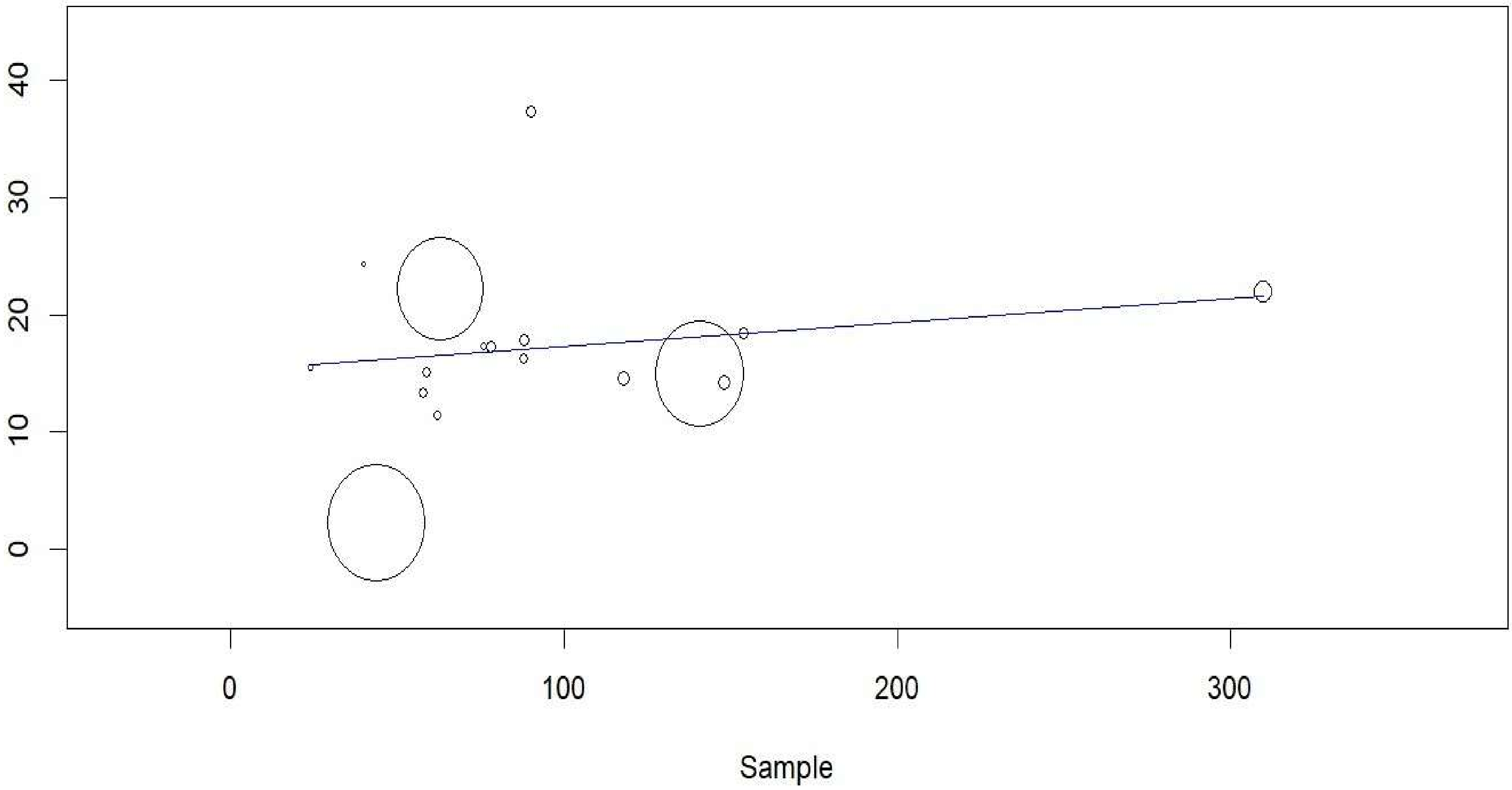

Supplementary Figure 5. Meta-regression analysis based on sample size

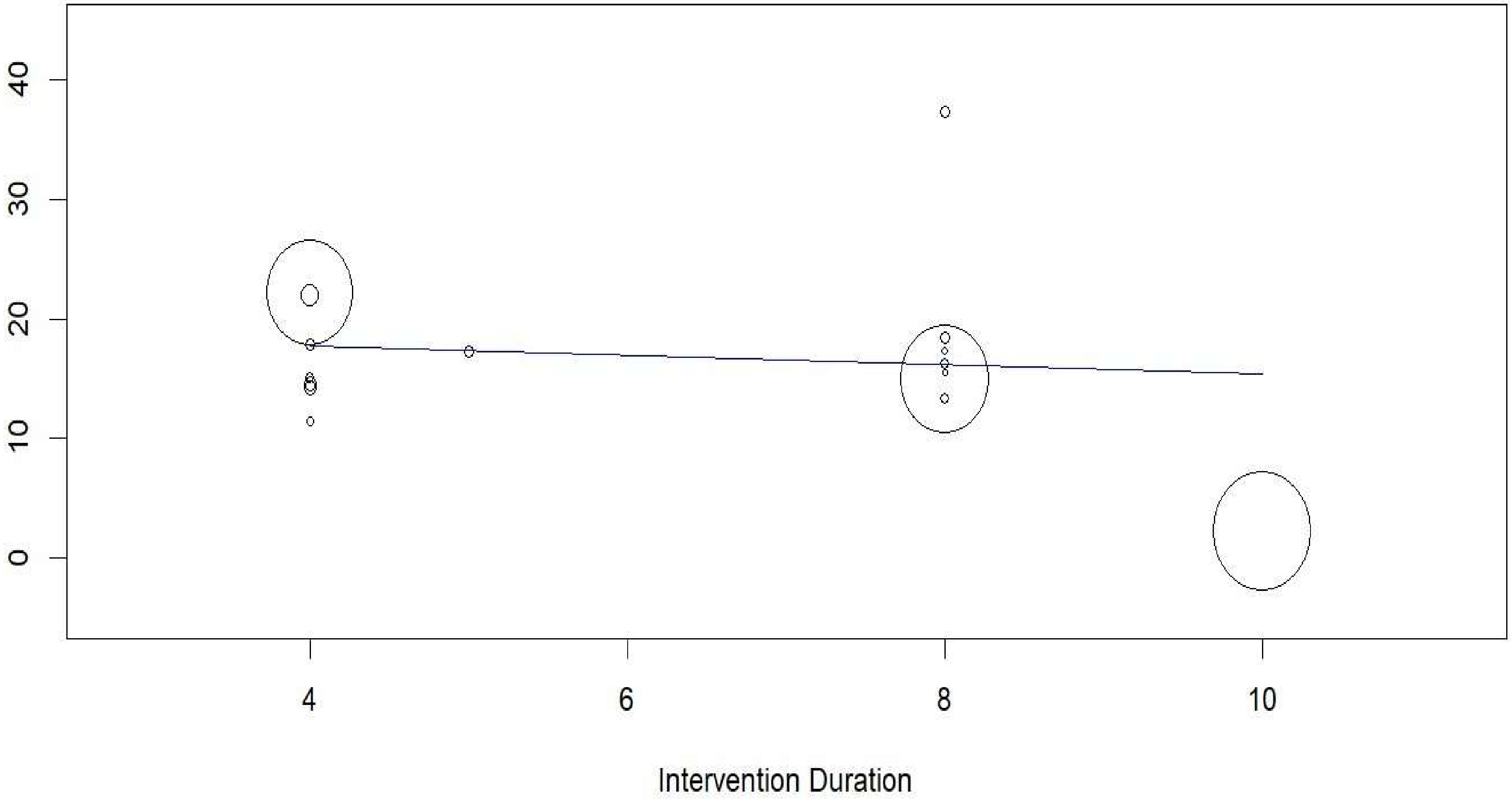

Supplementary Figure 6. Meta-regression analysis based on Intervention duration

Supplementary References

1. Xiong, Y. et al. The efficacy of digital mindfulness-based stress reduction on perceived stress, mindfulness, and self-compassion among Chinese college students: A randomized controlled trial. *Front. Psychiatry* **16**, 1644370 (2025). https://doi.org/10.3389/fpsyt.2025.1644370
2. Min, J. et al. Neurofeedback-assisted mindfulness training for employees via a mobile app: Randomized controlled trial. *JMIR Mhealth Uhealth* **11**, e42851 (2023). https://doi.org/10.2196/42851
3. Gallo, L. et al. Mindfulness-based relapse prevention (MBRP) for university students: Randomized controlled trial. *Int. J. Ment. Health Syst.* **17**, 32 (2023). https://doi.org/10.1186/s13033-023-00592-0
4. Boden, H. et al. Mindfulness applications: Can they serve as a stress, anxiety, and burnout reduction tool in orthopaedic surgery training? A randomized control trial. *JBJS Open Access* **8**, e22.00173 (2023). https://doi.org/10.2106/JBJS.OA.22.00173
5. Xu, H. G., Eley, R., Kynoch, K. & Tuckett, A. Effects of mobile mindfulness on emergency department work stress: A randomised controlled trial. *Emerg. Med. Australas.* **34**, 176–185 (2022). https://doi.org/10.1111/1742-6723.13836
6. Bartlett, L. et al. A randomized controlled trial of a workplace mindfulness program (Smiling Mind) to improve employee stress, well-being, and productivity. *JMIR Mhealth Uhealth* **10**, e30272 (2022). https://doi.org/10.2196/30272
7. Loh, K. J., Othman, A. & Phang, C. K. The effects of a brief mindfulness intervention on mindfulness, stress and emotional intelligence in medical students. *Educ. Med. J.* **14**, 1–24 (2022). https://doi.org/10.21315/eimj2022.14.2.1
8. Ritvo, P. et al. A mindfulness-based intervention for student depression, anxiety, and stress: Randomized controlled trial. *JMIR Ment. Health* **8**, e23491 (2021). https://doi.org/10.2196/23491
9. Sousa, G. M. et al. Brief mindfulness-based training and mindfulness trait attenuate psychological stress in university students: A randomized controlled trial. *BMC Psychol.* **9**, 21 (2021). https://doi.org/10.1186/s40359-021-00520-x
10. Ameli, R. et al. Effect of a brief mindfulness-based program on stress in health care professionals: A randomized clinical trial. *JAMA Netw. Open* **3**, e201266 (2020). https://doi.org/10.1001/jamanetworkopen.2020.1266
11. Huberty, J. et al. Efficacy of the mindfulness meditation mobile app ‘Calm’ to reduce stress among college students: Randomized controlled trial. *JMIR Mhealth Uhealth* **7**, e14273 (2019). https://doi.org/10.2196/14273
12. Lin, L. et al. The effects of a modified mindfulness-based stress reduction program for nurses: A randomized controlled trial. *Workplace Health Saf.* **67**, 111–122 (2019). https://doi.org/10.1177/2165079918801633

13. Champion, L., Economides, M. & Chandler, C. The efficacy of a brief app-based mindfulness intervention on psychosocial outcomes in healthy adults: A pilot randomised controlled trial. *PLoS One* **13**, e0209482 (2018). https://doi.org/10.1371/journal.pone.0209482

14. Querstret, D. et al. Mindfulness-based stress reduction and mindfulness-based cognitive therapy for occupational stress: A randomized controlled trial. *Mindfulness* **9**, 1478–1491 (2018). https://doi.org/10.1007/s12671-018-0901-8

15. Yang, E., Schamber, E., Meyer, R. M. L. & Gold, J. I. Happier healers: Randomized controlled trial of mobile mindfulness for stress management. *J. Altern. Complement. Med.* **24**, 505–513 (2018). https://doi.org/10.1089/acm.2015.0301

16. Ireland, M. J. et al. A randomized controlled trial of mindfulness to reduce stress and burnout among intern medical practitioners. *Med. Teach.* **39**, 409–414 (2017). https://doi.org/10.1080/0142159X.2017.1294749

17. Erogul, M., Singer, G., McIntyre, T. & Stefanov, D. Abridged mindfulness intervention to support wellness in first-year medical students: A randomized controlled trial. *Teach. Learn. Med.* **26**, 350–356 (2014). https://doi.org/10.1080/10401334.2014.945025
